# Integrated safety in tocilizumab clinical trials

**DOI:** 10.1186/ar3455

**Published:** 2011-09-01

**Authors:** Michael H Schiff, Joel M Kremer, Angelika Jahreis, Emma Vernon, John D Isaacs, Ronald F van Vollenhoven

**Affiliations:** 1 Rheumatology Division, University of Colorado School of Medicine, 5400 South Monaco Street, Greenwood Village, CO 80111, USA; 2 Center for Rheumatology, Albany Medical College, State University of New York, 1367 Washington Avenue, Albany, NY 12206, USA; 3 Product Development Inflammation, Genentech, Inc., 1 DNA Way, South San Francisco, CA 94080, USA; 4 PDBB-Biostatistics, Roche Products Ltd., Hexagon Place, 6 Falcon Way, Shire Park, Welwyn Garden City, UK AL7 1TW; 5 Institute of Cellular Medicine, Musculoskeletal Research Group, Newcastle University and Freeman Hospital, Catherine Cookson Building, Framlington Place, Newcastle upon Tyne, NE2 4HH, UK; 6 Rheumatology Department, Karolinska Institute, Solnavägen 1, Solna, Alfred Nobels Allé 8, Huddinge, SE-171 77 Stockholm, Sweden

## Abstract

**Introduction:**

The efficacy and safety of tocilizumab in patients with rheumatoid arthritis have been evaluated in a comprehensive phase 3 program. Patients from these randomized trials could receive tocilizumab treatment in open-label extension trials. Here, the long-term safety profile of tocilizumab, using pooled data from all of these trials, is reported.

**Methods:**

Cumulative safety data (as of February 6, 2009) from five core phase 3 trials, two ongoing extension trials, and one clinical pharmacology study were analyzed. Two patient populations were evaluated: an all-control population (*n *= 4,199), which included all patients randomly assigned in the placebo-controlled portions of the five core studies, and an all-exposed population (*n *= 4,009), which included patients from any of the eight studies who received at least one dose of tocilizumab.

**Results:**

Total exposure to tocilizumab was 8,580 patient years (PY), and total duration of observation was 9,414 PY. Overall adverse event (AE) and serious AE (SAE) rates were 278.2/100 PY and 14.4/100 PY, respectively. These events included serious infections (4.7/100 PY), opportunistic infections (0.23/100 PY), gastrointestinal perforations (0.28/100 PY), malignancy (1.1/100 PY), myocardial infarction (0.25/100 PY), and stroke (0.19/100 PY). The rates of SAEs and serious infections were stable over time; no increase with prolonged exposure was noted.

**Conclusions:**

The longer-term safety profile of tocilizumab (mean treatment duration, 2.4 years) is consistent with that observed in the phase 3 studies (duration up to 1 year).

## Introduction

Biologic agents that target tumor necrosis factor (TNF), B cells, T cells, or, most recently, interleukin-6 (IL-6) have emerged as effective treatments for patients with rheumatoid arthritis (RA). As with any new approach, evaluation of the safety profile associated with a particular treatment is critical.

Tocilizumab, a humanized monoclonal antibody that binds to both soluble and membrane-expressed IL-6 receptors, thereby blocking IL-6-mediated proinflammatory signaling, has one of the most comprehensive phase 3 clinical trial programs for biologicals in RA. In combination with disease-modifying antirheumatic drugs (DMARDs), tocilizumab improved signs and symptoms of RA [1-3] and inhibited radiographic progression of RA [4] in patients with inadequate responses to DMARDs or TNF inhibitors. Compared with methotrexate monotherapy, tocilizumab monotherapy also was significantly more effective in patients who had not been exposed to methotrexate or for whom methotrexate had not previously failed [5]. Overall, the onset of tocilizumab clinical benefit is rapid, and efficacy is sustained over time; reduced levels of inflammatory markers are observed as early as 14 days after the start of treatment [1-3,5,6].

Although the safety profile of tocilizumab was evaluated in each clinical trial, integrated data across all phase 3 studies [1-5] provide a more comprehensive picture of tocilizumab safety. Here we report pooled tocilizumab safety data and compare them with those of a control group from the RA phase 3 studies. Patients who participated in the randomized, placebo-controlled trials could continue to receive tocilizumab treatment in open-label extensions; therefore, this report includes long-term tocilizumab safety data not previously reported. We describe the longer-term safety profile of tocilizumab from these phase 3 studies and open-label extensions.

## Materials and methods

### Data sources and patient populations

**Figure 1:**
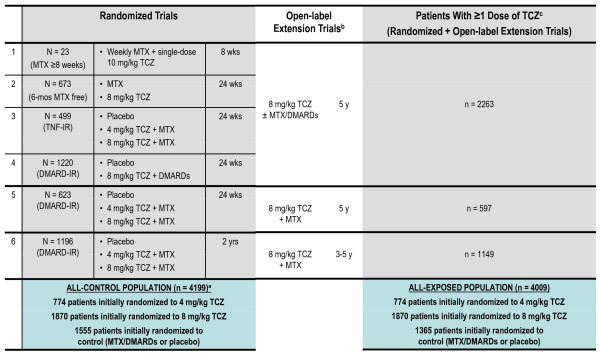
**Summary of clinical trials and patients in the all-exposed population**. ^a^Does not include patients from study 1; ^b^Extension studies are ongoing; most patients receive 8 mg/kg TCZ + MTX/DMARDs; ^c^All patients who received TCZ treatment; from their first dose (either in a core or an extension study) up to a cutoff date of February 6, 2009. DMARD-IR, inadequate responder to disease-modifying antirheumatic drugs; MTX, methotrexate; study 1, clinical pharmacology study; study 2, AMBITION; study 3, RADIATE; study 4, TOWARD; study 5, OPTION, study 6, LITHE; TCZ, tocilizumab; TNF-IR, inadequate responder to tumor necrosis factor inhibitor.

Included in this analysis are cumulative safety data from five core phase 3 clinical trials:

tOcilizumab Pivotal Trial in methotrexate Inadequate respONders (OPTION) [1], Actemra (Roche; Nutley, NJ, USA) versus Methotrexate double-Blind Investigative Trial In mONotherapy (AMBITION) [5] (including the double-blind transition phase), Research on Actemra Determining efficacy after Anti-TNF failurEs (RADIATE) [2], Tocilizumab in cOmbination With traditional DMARD therapy (TOWARD) [3], and tociLIzumab safety and THE prevention of structural joint damage (LITHE) [4] (including the ongoing open-label extension phase). Data also are included from the ongoing extension trials GROWTH95 and GROWTH96 and from a clinical pharmacology study [7] (Figure 1). The data cutoff date for inclusion in this analysis was February 6, 2009. Data that were corrected after the cutoff date are reported as corrected data. Given the relatively similar designs, populations, and data collection methods of the studies, individual patient data were pooled rather than weighted by study in a meta-analysis.

The *all-control population *included all patients randomly assigned in the five core studies. Data were included from double-blind phases of each core study, from randomization until the first change in treatment regimen (either to rescue therapy with tocilizumab or on entering the extension studies, including the open-label LITHE extension) or until 2 years of treatment.

The *all-exposed population *included all patients who received tocilizumab treatment (from their first tocilizumab dose (either in a core or an extension study) up to a cutoff date of February 6, 2009). Mean treatment duration was 2.4 years, and median treatment duration was 2.6 years (range, 0.0 to 4.1 years). Patients entered GROWTH 95 through August 2005 until February 2007 and GROWTH 96 through September 2005 until February 2008; both studies are ongoing. Patients continued background DMARD therapy unless adjustments or interruptions were required for safety reasons. Patients who received placebo during the controlled studies and who subsequently chose to enter the long-term extension studies were given tocilizumab 8 mg/kg once every 4 weeks.

### Dose modifications

Tocilizumab doses could be withheld or reduced on the occurrence of certain adverse events (AEs), specifically severe or frequent infections; significant elevations of alanine aminotransferase (ALT), aspartate aminotransferase (AST), or bilirubin; or decreases in absolute neutrophil count (ANC) or platelet count. In the placebo-controlled studies, dose modification was limited to skipping or interrupting an infusion of tocilizumab/placebo. In the long-term extension studies, a patient's tocilizumab dose could be decreased from 8 mg/kg to 4 mg/kg or skipped.

Dose and route of administration of methotrexate or other nonbiologic DMARDs, nonsteroidal anti-inflammatory drugs (NSAIDs), and oral corticosteroids were maintained in accordance with study entry through the placebo-controlled period of the trials. However, reductions in these treatments were allowed as clinically required for safety reasons.

### Safety and laboratory assessments

General safety measures, including AEs, serious AEs (SAEs), AEs leading to treatment discontinuation, and deaths were recorded. An AE was defined as any untoward medical occurrence in a patient who had been administered study treatment; a causal relation with the treatment was not necessary. An SAE was defined as any event that fulfilled regulatory seriousness criteria, including events leading to hospitalization, persistent or significant disability, medically significant events, or death. Multiple occurrences of the same AE in individual patients were counted once. Events of special interest (that is, serious infections; opportunistic infections, including tuberculosis (TB); gastrointestinal (GI) perforations; malignancies; myocardial infarction, and stroke; and anaphylactic reactions) are described later. Infections, opportunistic infections, malignancies, myocardial infarction, and stroke summaries were each based on a standardized group of preferred terms from the Medical Dictionary for Regulatory Activities (MedDRA); in these summaries, multiple occurrences of the same AE in individual patients were counted to assess overall event rates. Neutrophil and platelet counts, hepatic transaminase (ALT and AST) levels, and lipid levels were monitored by routine laboratory collections at scheduled visits (see Laboratory assessments later).

### Exposure to tocilizumab

The extent of tocilizumab exposure was calculated by assigning 28 days to each infusion received. Total duration of observation was defined as the time from the first tocilizumab dose to the time of the last safety observation, regardless of whether this observation was reported within 28 days of the last tocilizumab dose. Total duration of observation was used as the denominator for patient-adjusted AE rates.

### Laboratory assessments

Fasting lipid levels, including total, low-density lipoprotein (LDL) and high-density lipoprotein (HDL) cholesterol and triglycerides, were measured at baseline; at weeks 6, 14, and 24; and every 12 weeks thereafter. ALT, AST, and bilirubin levels were measured at baseline; at weeks 2, 4, 6, 8, 12, 14, 16, 20, and 24; and every 12 weeks thereafter. Complete blood counts (hemoglobin, hematocrit, red blood cells, white blood cells, and differential (absolute and percentage) and platelets) were performed at baseline; at weeks 2, 4, 6, 8, 12, 14, and 16; and every 4 weeks thereafter in the core studies. Complete blood counts were performed at baseline; at weeks 2, 4, 6, 8, and 12; and every 4 weeks thereafter in the extension studies. Laboratory measurements occurred before tocilizumab infusions, and investigators were unaware of test results at the time of infusion. Depending on the trial, it was either recommended or mandated that tocilizumab be permanently discontinued in any patient with the following laboratory values: a single ALT or AST measurement ≥ 5× the upper limit of normal (ULN) or two measurements ≥ 3× ULN at two consecutive study visits, a single total bilirubin level ≥ 2× ULN, or a neutrophil count < 0.5 × 10^9^/L. Tocilizumab treatment was withheld in any patient with an ALT or AST level ≥ 3× ULN until the level reached < 3× ULN. For liver enzyme assessments, the controlled, double-blind population was used because the monotherapy groups were specifically separated with regard to these measures for this population. Data were included up to only 6 months; patients randomly assigned to placebo for 8 weeks followed by tocilizumab for 16 weeks in a substudy of AMBITION were not included.

## Results

### Patient populations

The all-control population consisted of all patients randomly assigned in the five core studies; data from the double-blind periods were collected from randomization until the first change in treatment regimen or until 2 years of treatment. This population (*n *= 4,199) included 1,555 patients randomly assigned to the control groups (placebo ± methotrexate or placebo ± other DMARD(s)); 774 patients randomly assigned to tocilizumab 4 mg/kg + DMARD; and 1,870 patients randomly assigned to tocilizumab 8 mg/kg + DMARD. Duration of observation was 825 patient years (PY) for the control group, 565 PY for the tocilizumab 4 mg/kg + DMARD group, and 1,194 PY for the tocilizumab 8 mg/kg + DMARD group.

For liver enzyme assessments, 6-month controlled data were included from the five core studies for 1,170 patients in the control group (placebo + DMARD(s)); 284 patients in the methotrexate monotherapy group; 774 patients in the tocilizumab 4 mg/kg + methotrexate group; 1,582 patients in the tocilizumab 8 mg/kg + DMARD group; and 288 patients in the tocilizumab 8 mg/kg monotherapy group.

The all-exposed population included 4,009 patients who received at least one tocilizumab dose. Within this population, most of the tocilizumab exposure was at the 8-mg/kg dose: duration of tocilizumab exposure was 8,580 PY (817 PY for tocilizumab 4 mg/kg; 7,761 PY for tocilizumab 8 mg/kg; and 2 PY for tocilizumab 10 mg/kg). Total duration of observation was 9,414 PY, which is used as the denominator for the patient-adjusted AE rates presented hereafter.

### Overall safety

In the all-control population, the overall rate of AEs was 339.0/100 PY in the control group, 358.0/100 PY in the tocilizumab 4-mg/kg group, and 381.6/100 PY in the tocilizumab 8-mg/kg group. Rates of infection were 105.8/100 PY in the control group, 115.7/100 PY in the tocilizumab 4-mg/kg group, and 112.7/100 PY in the tocilizumab 8-mg/kg group The rate of SAEs was similar across the three treatment groups (control, 14.4/100 PY; tocilizumab 4 mg/kg, 13.6/100 PY; tocilizumab 8 mg/kg, 14.5/100 PY). SAEs reported at a rate of 0.3 or more per 100 PY in any group within the all-control population are shown in Table 1. Infections were the most frequently reported type of AE and SAE.

**Table 1:** Serious adverse events reported at a rate of ≥ 0.3 per 100 patient-years in any group (all-control population)

	All-control population *n *= 4,199		
	Control *n *= 1,555	Tocilizumab 4 mg/kg + DMARDs *n *= 774	Tocilizumab 8 mg/kg + DMARDs *n *= 1,870
Rate per 100 PY (number of events)			
Pneumonia	0.6 (5)	0.7 (4)	0.9 (11)
Cellulitis	0.2 (2)	--	0.9 (11)
Gastroenteritis	0.2 (2)	0.5 (3)	0.1 (1)
Urinary tract infection	0.5 (4)	0.2 (1)	0.1 (1)
Sepsis	0.1 (1)	0.4 (2)	0.2 (2)
Herpes zoster	0.1 (1)	--	0.3 (4)
Fall	0.1 (1)	--	0.3 (4)
Pulmonary embolism	0.2 (2)	--	0.3 (3)
Basal cell carcinoma	0.1 (1)	0.4 (2)	0.1 (1)
Spinal compression fracture	0.1 (1)	--	0.3 (3)
Coronary artery disease	--	0.2 (1)	0.3 (3)
Back pain	0.1 (1)	--	0.3 (3)
Rheumatoid arthritis	0.4 (3)	--	--
Gastroenteritis viral	0.1 (1)	0.4 (2)	--
Prostate cancer	0.1 (1)	0.4 (2)	--
Neutropenia	--	0.4 (2)	0.1 (1)
Syncope	--	0.4 (2)	--
Tendon rupture	--	0.4 (2)	--
Interstitial lung disease	--	0.4 (2)	--
Anaphylactic reaction	--	0.4 (2)	--

DMARD, disease-modifying antirheumatic drug; PY, patient-years.

In the all-exposed population, the AE rate was 278.2/100 PY. The most frequently reported types of AEs were infections (81.0/100 PY), usually upper respiratory tract infections and nasopharyngitis. GI disorders (usually diarrhea and nausea) occurred at a rate of 36.4/100 PY. The rate of AEs leading to discontinuation was 5.8/100 PY. Rates of AEs, AEs related to treatment, and AEs leading to discontinuation were highest during the first 6 months of tocilizumab treatment and decreased thereafter (Table 2). The rate of SAEs, which did not increase with prolonged exposure, was 14.4/100 PY.

**Table 2:** Rates of adverse events by duration of observation in the all-exposed population

	All-exposed population *n *= 4,009				
	Overall	≤ 6 months	7-12 months	13-18 months	> 18 months
Total duration, PY	9,414	1,805	1,664	1,542	4,404
AEs, rate/100 PY					
Any^a ^	278.2	441.1	340.4	284.7	245.5
Severe^a, b^	18.0	24.0	20.7	17.3	15.3
Treatment related^a, c^	112.8	214.7	141.0	106.4	85.2
Led to withdrawal^a^	5.8	11.5	6.7	4.9	3.5
Led to dose modification/ interruption^a^	36.6	41.3	40.2	37.3	37.8
SAEs, rate/100 PY					
Any^a^	14.4	15.9	15.1	13.9	14.0
Treatment related^c^	5.0	6.0	5.1	4.0	4.9

AE, adverse event; PY, patient-years; SAE, serious AE. ^a^Multiple occurrences of the same AE in one individual are counted once in each period. ^b^Intensity of AEs graded by the investigator as mild, moderate, or severe. ^c^Related AEs are those considered by the investigator to be remotely, possibly, or probably related to study drug.

The rate of death in the all-control population was 0.73/100 PY in the control group, 0/100 PY in the tocilizumab 4-mg/kg group, and 0.75/100 PY in the tocilizumab 8-mg/kg group. In the all-exposed population, the rate of death was 0.53/100 PY (50 cases). Most frequently reported causes of death were cardiac events (13 cases), serious infections (12 cases), and malignancies (eight cases).

### Clinical events and laboratory abnormalities leading to treatment discontinuation or dose reduction

In the all-control population, the rate of AEs leading to treatment discontinuation was 6.9/100 PY in the control group and 10.1/100 PY and 10.2/100 PY in the tocilizumab 4-mg/kg and 8-mg/kg groups, respectively. Infections were the most common AEs leading to discontinuation in the control group (1.3/100 PY), and liver function test abnormalities were the most common AEs leading to discontinuation in the tocilizumab groups (2.5/100 PY, 4-mg/kg group; 2.8/100 PY, 8-mg/kg group). The most common AEs leading to dose modification or interruption were infections (12.2/100 PY (control group), 15.8/100 PY (tocilizumab 4-mg/kg group), and 15.2/100 PY (tocilizumab 8-mg/kg group)).

In the all-exposed population, the most frequently reported AEs leading to treatment discontinuation were investigations (1.3/100 PY, primarily hepatic transaminase elevations) and infections (1.1/100 PY, primarily pneumonia and cellulitis). Fifty-six patients withdrew because of malignant neoplasms (including one patient with nonmelanoma skin cancer), and 43 patients withdrew because of GI disorders. The most common AEs leading to reductions in tocilizumab dose from 8 to 4 mg/kg were laboratory abnormalities (primarily increased aminotransferases). Of the 257 AEs (in 217 patients) managed by dose reduction, 247 (96%) resulted in continuation of tocilizumab therapy.

### Serious infections

In the all-control population, rates of serious infections were 3.5/100 PY for the control group, 3.5/100 PY for the tocilizumab 4-mg/kg group, and 4.9/100 PY for the tocilizumab 8-mg/kg group. Higher rates of serious infection, regardless of whether infections were in the control or the tocilizumab groups, were associated with increased age, body mass index ≥ 30, previous use of a TNF inhibitor, and history of chronic (obstructive or restrictive) pulmonary disease or diabetes (for control and tocilizumab 8 mg/kg) (Table 3). The rate of serious pneumonia was higher in patients with (4.9/100 PY) than without (0.8/100 PY) chronic pulmonary disease.

**Table 3:** Rates of serious infections (overall and by baseline characteristics) in the all-control population

	Exposure, PY	Control *n *= 1,555	Exposure, PY	Tocilizumab 4 mg/kg + DMARDs *n *= 774	Exposure, PY	Tocilizumab 8 mg/kg + DMARDs *n *= 1,870
Rate/100 PY^a ^(number of events^b^)						
Overall	824.6	3.5 (29)	564.6	3.5 (20)	1,194.1	4.9 (59)
Rate/100 PY^a ^(95% CI^a^), (number of events^b^)						
Age, years						
< 50	340.2	1.5 (0.5, 3.4), (5)	244.2	1.6 (0.5, 4.2), (4)	431.3	1.9 (0.8, 3.7), (8)
≥ 50-64	354.2	3.7 (2.0, 6.3), (13)	229.9	3.5 (1.5, 6.9), (8)	572.8	5.9 (4.1, 8.3), (34)
≥ 65	130.1	8.5 (4.2, 15.1), (10)	90.6	8.8 (3.8, 17.4), (8)	189.9	9.0 (5.2, 14.3), (17)
BMI,^c ^kg/m^2^						
< 18.5	29.2	--	9.6	--	31.3	16.0 (5.2, 37.3), (5)
18.5-24.9	282.1	2.8 (1.2, 5.6), (7)	209.4	3.8 (1.7, 7.5), (8)	414.4	2.7 (1.3, 4.8), (11)
25-29.9	275.6	3.6 (1.7, 6.7), (10)	187.2	3.2 (1.2, 7.0), (6)	412.5	5.3 (3.3, 8.1), (22)
≥ 30	234.2	4.7 (2.3, 8.4), (11)	154.7	3.9 (1.4, 8.4), (6)	327.8	6.1 (3.7, 9.4), (20)
Background corticosteroid use						
Yes	570.5	3.3 (2.0, 5.2), (18)	426.6	4.7 (2.9, 7.2), (20)	788.3	5.3 (3.8, 7.2), (42)
No	254.0	3.9 (1.9, 7.2), (10)	138.0	--	405.8	4.2 (2.4, 6.7), (17)
Previous TNF-α inhibitor therapy						
Yes	134.6	6.7 (3.1, 12.7), (9)	114.4	5.3 (1.9, 11.4), (6)	200.1	8.5 (5.0, 13.6), (17)
No	690.0	2.9 (1.8, 4.5), (19)	450.2	3.1 (1.7, 5.2), (14)	994.0	4.2 (3.1, 5.7), (42)
Chronic pulmonary disease						
Yes	38.9	10.3 (2.8, 26.3), (3)	28.4	10.6 (2.2, 30.8), (3)	54.1	7.4 (2.0, 18.9), (4)
No	785.6	3.2 (2.1, 4.7), (25)	536.2	3.2 (1.9, 5.1), (17)	1,140.0	4.8 (3.6, 6.3), (55)
Serious infections						
Yes	--	10.3 (2.8, 26.3), (3)	--	10.6 (2.2, 30.8), (3)	--	7.4 (2.0, 18.9), (4)
No	--	3.2 (2.1, 4.7), (25)	--	3.2 (1.9, 5.1), (17)	--	4.8 (3.6, 6.3), (55)
Serious pneumonia						
Yes	--	7.7 (1.6, 22.5)	--	3.5 (0.1, 19.6)	--	5.6 (1.1, 16.2)
No	--	0.4 (0.1, 1.1)	--	0.9 (0.3, 2.2)	--	0.9 (0.4, 1.6)
Diabetes						
Yes	66.6	7.5 (2.4, 17.5), (5)	54.1	1.9 (0.1, 10.3), (1)	99.4	12.1 (6.2, 21.1), (12)
No	758.0	3.2 (2.0, 4.7), (23)	510.5	3.7 (2.2, 5.8), (19)	1,094.7	4.3 (3.2, 5.7), (47)

BMI, body mass index; CI, confidence interval; DMARD, disease-modifying antirheumatic drug; PY, patient-years; TNF, tumor necrosis factor.

^a^Multiple occurrences of the same adverse event (AE) in one individual are counted. ^b^Multiple occurrences of the same AE in one individual are counted only once. ^c^Twenty-two patients did not have BMI values.

**Figure 2:**
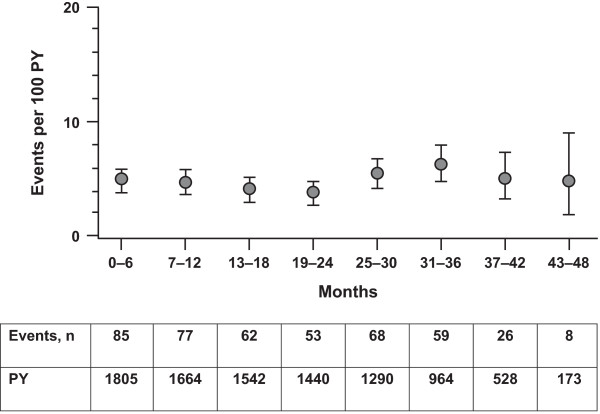
**Rates of serious infections (95% confidence intervals) in the all-exposed population by 6-month periods**. PY, patient-years. Multiple occurrences of the same adverse event in a patient in a 6-month period were counted as individual events.

In the all-exposed population, the rate of serious infections was 4.7/100 PY, and the most frequent events were pneumonia (1.0/100 PY), gastroenteritis, and urinary tract infections. The rate of serious infections remained relatively stable with continued tocilizumab treatment (Figure 2). However, rates increased with age: 2.9/100 PY for patients younger than 50, 5.1/100 PY for patients 50 to 64, and to 8.1/100 PY for patients 65 and older.

### Opportunistic infections

Overall, 22 opportunistic infections (0.23/100 PY), 14 of which were serious, were reported in 20 patients in the all-exposed population. The types of opportunistic infections included TB (eight cases), candidiasis (systemic, esophageal, GI, osteomyelitis; six cases), fungal infections (microorganism not specified; three cases), other mycobacterial infections (*Mycobacterium avium *and nonspecified; one case each), and *Pneumocystis jiroveci *pneumonia and cryptococcal pneumonia (one case each). All opportunistic infections occurred in patients treated with tocilizumab 8 mg/kg, except for *P. jiroveci *pneumonia, which occurred in a patient treated with tocilizumab 4 mg/kg. No opportunistic infections were reported in the control group.

#### Tuberculosis

Investigators were instructed to screen for TB according to local guidelines for RA patients starting biologic therapy. Chest radiographs were obtained for all patients at the screening visit. In the all-exposed population, eight cases of infections with *M. tuberculosis *(see previous paragraph) were reported in seven patients; for one patient, tuberculosis pleurisy and TB were reported during the same episode. None of the patients with available data reported a relevant medical history, history of TB, or previous known exposure to TB. All patients were treated with concomitant DMARDs. Pulmonary TB was reported in four patients, tuberculosis pleurisy in three, and tuberculosis (not otherwise specified) in one. The patients with pulmonary TB were from Thailand, Spain, South Africa, and Peru, and had symptoms of productive cough, hemoptysis, pleuritic pain, and fatigue, or chest radiograph findings suggestive of TB. Tuberculosis pleurisy was described in three patients from Singapore, Brazil, and Peru. *M. tuberculosis *was confirmed by polymerase chain reaction in a patient who had a foot abscess and negative findings on the tuberculin test in Mexico.

### Gastrointestinal perforations

Hospital, surgical, and discharge summaries from cases of GI perforation were medically reviewed. In the all-control population, diverticular perforation occurred in one patient in the tocilizumab 4-mg/kg group (0.2/100 PY) and in two patients in the tocilizumab 8-mg/kg group (0.2/100 PY). In the all-exposed population, 26 cases (0.28/100 PY) were identified as perforations, including the following diagnoses: abscesses (four cases), appendicitis (two cases), diverticulitis (three cases), fistula (one case), and GI perforations (16 cases, including 11 diverticular perforations). Eighteen of these events occurred in the colon, of which 16 occurred in patients with underlying diverticulitis. Most (65%) patients with GI perforations were women, with a mean age of 58.5 years at trial enrollment. Three patients with GI perforations died--one of postprocedural esophageal perforation, one of perforation associated with an upper GI bleed, and one of complications of diverticular perforation.

### Malignancies

Overall rates of malignancy (including nonmelanoma skin cancer) in the all-control population were 0.7/100 PY, 1.4/100 PY, and 0.7/100 PY in the control, tocilizumab 4-mg/kg and tocilizumab 8-mg/kg groups, respectively. In the all-exposed population, the overall rate of malignancy was 1.1/100 PY. Rates were 0.6/100 PY for solid cancer, 0.4/100 PY for nonmelanoma skin cancers, < 0.1/100 PY for hematologic/lymphatic cancers, and < 0.1/100 PY for other cancers. Overall rates of malignancy remained stable over time, and no increase was found with prolonged exposure. The overall rate of malignancy of 1.1/100 PY in this analysis of the all-exposure population was comparable to the rate of 1.3/100 PY observed in a contemporary large United States cohort of RA patients (41,912 PY) in which 62% of patients were treated with TNF inhibitors [8]. The standardized incidence ratio for all cancer types (excluding nonmelanoma skin cancers) compared with the US Surveillance and Epidemiology End Results database was 0.80 (95% CI, 0.78, 0.82) in the current analysis, indicating no statistically significant difference compared with the general population.

### Myocardial infarction and stroke

The overall number of cardiovascular (CV) events that occurred during the placebo-controlled period of the studies was low (Table 4). In the all-control population, the myocardial infarction rate was 0.49/100 PY in the control group, 0.18/100 PY in the tocilizumab 4-mg/kg group, and 0.17/100 PY in the tocilizumab 8-mg/kg group. The rate of strokes was 0.24/100 PY in the control group, 0/100 PY in the tocilizumab 4-mg/kg group, and 0.33/100 PY in the tocilizumab 8-mg/kg group (Table 4). In the all-exposed population, the myocardial infarction rate was 0.25/100 PY (95% CI, 0.16, 0.38), and the rate of stroke was 0.19/100 PY (95% CI, 0.11, 0.30) at 9,414 PY of exposure. Rates were assessed by 6-month intervals, and no apparent trend toward an increase was noted over time. Two CV events occurred within 10 days of starting tocilizumab dosing: acute coronary syndrome in a patient with high-grade, three-vessel disease, which necessitated a four-vessel coronary artery bypass on day 13; and carotid artery stenosis in a patient with 90% occlusion of the right carotid artery, as assessed by ultrasound on day 18.

**Table 4:** Rates of myocardial infarction and stroke in the all-control population

	All-control population *n *= 4,199		
	Control *n *= 1,555	Tocilizumab 4 mg/kg + DMARDs *n *= 774	Tocilizumab 8 mg/kg + DMARDs *n *= 1,870
Rate/100 PY (95% CI), (number of events)			
Myocardial infarction	0.49 (0.13, 1.24), (4)	0.18 (0.00, 0.99), (1)	0.17 (0.02, 0.61), (2)
Stroke	0.24 (0.03, 0.88), (2)	0.0	0.33 (0.09, 0.86), (4)

CI, confidence interval; DMARD, disease-modifying antirheumatic drug; PY, patient-years. Multiple occurrences of the same adverse event in individual patients were counted.

### Anaphylactic reactions

In the all-control population, three anaphylactic reactions were reported in the tocilizumab 4-mg/kg group (0.5/100 PY), and one was reported in the tocilizumab 8-mg/kg group (0.1/100 PY). In the all-exposed population, eight clinically significant anaphylactic reactions, including the four cases from the all-control population, were reported. Symptoms included urticaria, nausea, vomiting, hypotension, hypotensive shock, and bronchospasm. All eight patients with anaphylaxis were required to discontinue tocilizumab treatment. Five events occurred in patients treated with tocilizumab 4 mg/kg, and three events occurred in patients treated with tocilizumab 8 mg/kg. Seven events occurred during the first four infusions of tocilizumab. One late event occurred after completion of the 16th infusion, 15 hours after tocilizumab infusion.

### Laboratory parameters

#### Neutrophil count

Blood samples were obtained immediately before each tocilizumab infusion. In the all-control population, decreases from baseline to week 2 in mean neutrophil count were noted in all three groups: 5.91 × 10^9^/L to 5.87 × 10^9^/L in the control group, 5.96 to 4.38 × 10^9^/L in the tocilizumab 4-mg/kg group, and 5.82 to 3.68 × 10^9^/L in the tocilizumab 8-mg/kg group. At week 4, mean neutrophil counts increased to 5.89 × 10^9^/L, 5.70 × 10^9^/L, and 4.31 × 10^9^/L, respectively; corresponding values at week 24 were 5.46 × 10^9^/L, 5.10 × 10^9^/L, and 3.83 × 10^9^/L.

Mean neutrophil count in the all-exposed population was 5.82 × 10^9^/L at baseline. From the first postdose assessment (week 2), mean neutrophil count remained relatively stable over time: for example, values at weeks 2, 4, and 24 were 3.85, 4.61, and 4.07 × 10^9^/L, respectively. Common toxicity criteria (CTC) grade 3 neutropenia (ANC 0.5 to < 1.0 × 10^9^/L) was reported in 4.1% of patients (165/3,992). CTC grade 4 (ANC < 0.5 × 10^9^/L) neutropenia while receiving tocilizumab was reported in 0.6% of patients (24/3,992); 16 of the 24 patients with CTC grade 4 neutropenia discontinued tocilizumab treatment. For 15 patients, further neutrophil data were available, and neutrophil counts returned to normal levels in all but one patient. This patient was enrolled in the clinical trial despite neutropenia at baseline. Of the 24 patients who had CTC grade 4 neutropenia while receiving tocilizumab, eight continued treatment. All eight experienced improvements in neutropenia--at the time of database cutoff, neutrophil counts recovered to within normal range in five patients, and neutropenia recovered to CTC grade 2 in three patients. No patient with CTC grade 4 neutropenia experienced a temporally associated serious infection. A single patient with CTC grade 3 neutropenia reported a serious infection (empyema) that occurred shortly after CTC grade 3 neutropenia became evident.

#### Platelet count

In the all-control population, mean platelet count was unchanged from baseline to week 2 in the control group (339 × 10^9^/L). However, decreases in mean platelet count occurred in the tocilizumab groups (337 × 10^9^/L at baseline to 259 × 10^9^/L at week 2 in the 4-mg/kg group; 334 × 10^9^/L at baseline to 249 × 10^9^/L at week 2 in the 8-mg/kg group). After week 2, mean platelet counts remained relatively unchanged. In the all-exposed population, 32 patients had thrombocytopenia (either ≥ 25 to < 50 × 10^9^/L platelets (grade 3, 13 patients) or < 25 × 10^9^/L platelets (grade 4, 19 patients)). One serious bleeding event (hemorrhagic stomatitis) occurred in a patient with CTC grade 4 thrombocytopenia. The 8-mg/kg dose was maintained, and the event resolved without sequelae.

#### Hemoglobin

In the all-control population, the mean hemoglobin level was 13.5 g/dl at baseline in all three groups. At weeks 2 and 6, levels were 13.3 g/dl in controls; 14.1 g/dl and 14.2 g/dl, respectively, in the tocilizumab 4-mg/kg group; and 14.1 g/dl and 14.4 g/dl, respectively, in the tocilizumab 8-mg/kg group. Mean baseline hemoglobin level in the all-exposed population was 13.4 g/dl. Subsequently, the level increased to 14.0 g/dl (week 2), 14.3 g/dl (week 6), and 14.8 g/dl (week 104). Approximately 34% of patients who previously had inadequate responses to DMARDs had hemoglobin values below the lower limit of normal at baseline. After treatment with tocilizumab, the greatest improvement in hemoglobin levels was observed in these patients, whose baseline hemoglobin values were below the lower limit of normal. In this subset, mean hemoglobin concentrations increased in the tocilizumab-treated group to within the lower limit of normal, whereas mean levels in the control group remained below the lower limit of normal.

#### Assessment of lipids

Fasting lipid levels were assessed during the tocilizumab development program. In the all-control population, mean LDL levels in the control, tocilizumab 4-mg/kg, and tocilizumab 8-mg/kg groups were 114 mg/dl (*n *= 1,404), 113 mg/dl (*n *= 690), and 113 mg/dl (*n *= 1,686), respectively, at baseline, and 117 mg/dl (*n *= 939), 127 mg/dl (*n *= 536), and 136 mg/dl (*n *= 1,419), respectively, at week 24. LDL cholesterol changes from < 130 mg/dl at baseline to ≥ 130 mg/dl at last observation were assessed in patients not receiving lipid-lowering agents; 14.2% (123/867) in the control group, 25.1% (106/423) in the tocilizumab 4-mg/kg group, and 33.2% (349/1,052) in the tocilizumab 8-mg/kg group experienced these elevations in LDL cholesterol levels.

In the all-exposed population, mean total cholesterol, LDL, HDL, and triglyceride levels increased from baseline to the first assessment after tocilizumab infusion at week 6 and remained at week 6 levels with continued tocilizumab treatment (Table 5). At baseline, 70.8% of patients had LDL levels < 130 mg/dl. This proportion decreased to 49.9% at week 24 and remained relatively constant throughout the trial (for example, 48.2% at week 104 and 45.9% at week 200). Overall, 456 patients in the all-exposed population started treatment with lipid-lowering medications at some point during the study. Assessment of the exact effect and the time course of the effect of lipid-lowering medications is difficult because of the wide range of times at which patients started taking these medications in the trials. Furthermore, initiation and dosing of lipid-lowering therapy were not standardized but were based on the discretion of the treating physician. However, after an initial increase in mean LDL level, a decrease to a level similar to that at baseline was observed in this subgroup: baseline (135 mg/dl), week 6 (167 mg/dl), week 52 (152 mg/dl), and week 104 (139 mg/dl) (Table 6).

**Table 5:** Lipid parameters^a ^in the all-exposed population (*n *= 4,009)

Parameter (mg/dl)	Baseline	Week 6	Week 52^b^	Week 104
Mean total cholesterol	196.2 (*n *= 3,714)	227.6 (*n *= 3,279)	222.9 (*n *= 1,312)	224.1 (*n *= 828)
Mean LDL cholesterol	114.2 (*n *= 3,633)	135.5 (*n *= 3,149)	133.5 (*n *= 1,274)	134.3 (*n *= 805)
Mean HDL cholesterol	56.9 (*n *= 3,656)	60.8 (*n *= 3,249)	60.0 (*n *= 1,301)	60.0 (*n *= 826)
Mean triglycerides	127.6 (*n *= 3,715)	160.4 (*n *= 3,280)	148.8 (*n *= 1,312)	148.8 (*n *= 828)
Patients starting lipid-lowering medication at any time up to week 104 during the trial (*n *= 456)				
Mean total cholesterol	223.3 (*n *= 415)	267.4 (*n *= 375)	247.7 (*n *= 154)	236.1 (*n *= 109)
Mean LDL cholesterol	135.1 (*n *= 402)	167.6 (*n *= 338)	152.9 (*n *= 142)	138.9 (*n *= 97)

HDL, high-density lipoprotein; LDL, low-density lipoprotein. ^a^Fasted samples only. ^b^Patient numbers declined after week 6 because patients' 12-week fasted lipid-analysis schedules might not have coincided with week 52, depending on when they started tocilizumab treatment (that is, some patients had their fasted lipid analysis at week 48 or week 56).

**Table 6:** Changes in ALT/AST values from normal at baseline to highest value in the all-control and in the all-exposed population

	Controlled, double-blind study population					All-exposed population
	**Tocilizumab 8 mg/kg monotherapy**, % (*n*) *n *= 288	**Methotrexate (control)**, % (*n*) *n *= 284	**Tocilizumab 4 mg/kg + DMARDs**, % (*n*) *n *= 774	**Tocilizumab 8 mg/kg + DMARDs**, % (*n*) *n *= 1,582	**DMARD monotherapy**, % (*n*) *n *= 1,170	**Tocilizumab**, % (n/n) *n *= 4,009^c^
ALT,^a ^*n *= normal at baseline	*n *= 269	*n *= 269	*n *= 706	*n *= 1,465	*n *= 1,080	
> 1-3× ULN > 3-5× ULN > 5× ULN	33.8 (91) 1.1 (3) 0.7 (2)	32.0 (86) 2.6 (7) 1.1 (3)	42.8 (302) 4.0 (28) 1.0 (7)	45.9 (672) 4.3 (63) 1.4 (20)	19.1 (206) 0.8 (9) 0.3 (3)	57.3 (2,112/3,696) 7.2 (267/3,696) 2.2 (83/3,696)
AST,^a ^*n *= normal at baseline	*n *= 283	*n *= 269	*n *= 743	*n *= 1,502	*n *= 1,123	
> 1-3× ULN > 3-5× ULN > 5× ULN	20.8 (59) 0.4 (1) 0.7 (2)	24.9 (67) 1.1 (3) 0.4 (1)	32.4 (241) 0.9 (7) --	38.8 (583) 1.5 (23) 0.2 (3)	14.5 (163) 0.3 (3) 0.1 (1)	51.4 (1,961/3,818) 2.6 (98/3,818) 0.6 (22/3,818)
Dose held^b^	8.0 (23)	9.9 (28)	2.5 (19)	2.5 (39)	0.7 (8)	10.3 (413/4,009)
Discontinued	0.3 (1)^b^	1.4 (4)^b^	1.3 (10)^b^	1.3 (21)^b^	0.2 (2)^b^	2.3 (91/4,002)

ALT, alanine aminotransferase; AST, aspartate aminotransferase; DMARD, disease-modifying antirheumatic drug; ULN, upper limit of normal. ^a^Percentages are based on number of patients with normal ALT (or AST) at baseline. ^b^Percentages are based on total treatment-group sample size. ^c^Excluding patients with missing values.

#### Transaminase elevations

Liver enzyme elevations were assessed in the controlled, double-blind periods of the original studies. Patients treated with monotherapy had less-pronounced liver enzyme elevations than did patients treated with combination therapy; 32% and 3.7% of patients treated with methotrexate monotherapy; 19.1% and 1.1% of patients treated with non-methotrexate DMARD monotherapy; and 33.8% and 1.9% of patients treated with tocilizumab 8-mg/kg monotherapy each had at least a single increase in ALT level from normal at baseline to > 1 to 3× ULN or > 3× ULN, respectively, as the highest value during the double-blind period. Further, 42.8% and 5.0% of patients treated with tocilizumab 4 mg/kg + DMARD, and 45.9% and 5.7% of patients treated with tocilizumab 8 mg/kg + DMARD, each had at least a single elevation in ALT level from normal at baseline to > 1 to 3× ULN or > 3× ULN, respectively, as their highest value during the double-blind period (Table 6). Changes in AST values were similar (Table 6).

In the all-exposed population, 9.5% and 3.1% of patients treated with tocilizumab had increases in ALT or AST levels from normal at baseline to > 3× ULN at any time up to the time of database lock. Overall, 1.6% of patients had tocilizumab dose reductions from 8 mg/kg to 4 mg/kg; 0.4% of patients had DMARD dose reductions; 9.3% of patients temporarily interrupted tocilizumab dosing; and 1% of patients had interrupted DMARD and tocilizumab dosing because of liver enzyme elevations > 3× ULN. Most patients continued therapy after dose reduction; 2.3% of patients withdrew from treatment because of elevated liver function tests (Table 6). In the all-control population, mean total bilirubin levels at baseline were 5 to 6 μmol/L (0.30 to 0.35 mg/dl) in all groups. Mean increases to week 4 in total and indirect bilirubin levels were ≤ 2 μmol/L (0.12 mg/dl) in all groups. These values remained stable during the long-term extension studies, and no clinically relevant effect was noted on direct bilirubin, albumin, alkaline phosphatase, or any other liver function parameter. Further, no evidence was seen of clinically significant hepatitis or drug-induced liver injury associated with transaminase elevations in patients treated with tocilizumab.

Liver biopsies were recommended but not required for patients whose liver-enzyme elevations did not normalize within 6 months of discontinuation from the study and were obtained at the investigators' discretion. Of the 11 liver biopsy samples evaluated by an independent hepatic pathology expert, steatohepatitis was present in nine patients. Seven of the nine patients had ≥ 1 clinical risk factor (excluding methotrexate use) for nonalcoholic steatohepatitis, including obesity, diabetes, and hyperlipidemia. The two patients with normal liver biopsy samples had no risk factors for liver disease and had no history of previous liver disease.

## Discussion

The safety profile of tocilizumab was extensively evaluated in five trials in patients with RA, and the pooled safety data from these phase 3 trials and their long-term extensions are described herein. The longer-term safety profile of tocilizumab (mean treatment duration, 2.4 years) is consistent with that observed in the phase 3 studies (treatment duration, up to 1 year). Notably, no increases in serious or treatment-related AEs were observed with prolonged exposure to tocilizumab. Rates of SAEs, including deaths, were similar to those observed in RA clinical trials for other biologic therapies [9], although direct comparison of rates may be inappropriate because of differences in study designs and populations. Further, data from this pooled safety report are consistent overall with the postmarketing trials and experience with tocilizumab.

Patients with RA are approximately twice as likely as the general population to acquire infections or serious infections [10,11]. This increased risk for infection is attributed to intrinsic disease-related factors (for example, immunologic abnormalities [12]), age, extra-articular manifestations, leukopenia, comorbidities, and treatment-related factors (for example, use of corticosteroids) [13]. In the current analysis, rates of serious infection in the control and tocilizumab-treated groups were higher in patients with preexisting pulmonary disease, diabetes, older age, or higher body mass index and in those concomitantly treated with steroids or previously treated with a TNF inhibitor.

Overall, infections were the most common AEs and SAEs reported during the studies included in this analysis. Compared with the control group, rates of infections were higher in the tocilizumab 8-mg/kg group than in the tocilizumab 4-mg/kg group. Eight cases of infections with TB were reported in seven patients, all of whom received tocilizumab 8 mg/kg. The most common serious infections involved the skin (cellulitis) and lung (pneumonia). Importantly, rates of serious infection did not increase with prolonged use of tocilizumab, and serious infections occurred at rates similar to those reported for RA patients treated with TNF inhibitors or other biologic agents [14,15]. Patients with RA treated with any biologic treatment, including tocilizumab, should be closely monitored for the development of infections, and--according to American College of Rheumatology Recommendations for Use of Nonbiologic and Biologic DMARDs [16]--treatment should not be started in a patient with an active infection. Recommendations also include routine TB screening for patients being considered for biologic therapy and clinical vigilance during long-term treatment.

In the current analysis, 26 cases (0.28/100 PY) of GI perforation, including three fatal perforations, were observed during treatment with tocilizumab. According to the results of a retrospective, claims-based study that evaluated 40,841 RA patients treated with biologic agents, methotrexate, oral glucocorticoids, and NSAIDs, GI perforation is an uncommon (0.11 hospitalizations with GI perforation per 100 PY) SAE among RA patients [17]. Oral glucocorticosteroid use and preexisting diverticulitis were independently associated risk factors for GI perforation. Overall, 70% of RA patients with GI perforation in the analysis had one or both of these risk factors. Therefore, RA patients who are treated with glucocorticoids or who have previously experienced diverticulitis should be considered at higher risk for GI perforation. Tocilizumab-treated patients with new-onset abdominal symptoms should be evaluated promptly for the early identification of GI perforation.

In this integrated summary of safety, eight clinically significant anaphylactic reactions to tocilizumab infusions were reported. Most of these events occurred with the 4-mg/kg tocilizumab dose and early during treatment. During tocilizumab infusion, treatment should be stopped immediately and appropriate medical management should be started if an anaphylactic or other serious hypersensitivity reaction occurs. Tocilizumab should be permanently discontinued in patients who experience these reactions.

Increases in hepatic transaminase (ALT and AST) levels were observed in some patients in the phase 3 trials [1-5]. These increases were generally mild and reversible. Increases in ALT or AST to either > 1 to 3× ULN or > 3× ULN in patients treated with tocilizumab were less common with tocilizumab monotherapy. Importantly, liver enzyme elevations were not associated with clinically apparent drug-induced liver injury. Because of the potential for increases in hepatic transaminase levels, these laboratory values should be monitored before and during tocilizumab therapy, and dose adjustments--including interruption or permanent discontinuation of tocilizumab or concomitant DMARD (particularly methotrexate)--should be considered if appropriate.

Finally, RA has been associated with an increased risk for CV disease and CV-related deaths [18-21], despite the paradoxically low lipid levels (total cholesterol, HDL, and LDL) seen with active disease and inflammation. Although the pathogenic mechanisms involved in accelerated CV disease in RA patients are complex and multifactorial, the underlying systemic inflammation has been postulated to promote accelerated atherosclerosis. This concept is supported by the fact that inflammatory markers such as CRP have been shown to be independent predictors of CV events in the general population [22,23], and the reduction of hsCRP by rosuvastatin in the JUPITER study reduced the incidence of major CV events [24]. Reducing the inflammatory burden of RA by lowering CRP and IL-6 levels may, therefore, reduce CV risk in RA patients [25-27]. In this pooled data set, rates of myocardial infarction and stroke were similar to those reported in epidemiologic studies of RA patients [18,28,29]. Aggressive prevention and treatment of traditional and nontraditional CV risk factors, including control of inflammation, immunologic disturbances, and metabolic changes, are of paramount importance in RA patients. Like patients without RA, patients with RA--particularly those actively treated--should be monitored and managed according to clinical guidelines (for example, the National Cholesterol Education Program). Lipid parameters should be assessed 4 to 8 weeks after the start of RA treatment and at 6-month intervals thereafter; the addition of lipid-lowering agents in patients with persistently elevated lipid levels should be considered.

In summary, this report provides an account of the safety profile of tocilizumab from phase 3 clinical trials and their respective open-label extensions. Results demonstrate that the longer-term safety profile of tocilizumab is consistent with that observed in phase 3 studies, with no increases in serious or treatment-related AEs on prolonged exposure to tocilizumab. Similar trial designs and data collection allowed the pooling of individual patient data and resulted in a very large RA randomized controlled trial data set with extensive long-term exposure. However, as with any randomized controlled trial, patients were assessed for strict entry criteria, and the observations were made in a clinical trial setting with rigorous patient monitoring and laboratory testing protocols. Several of the laboratory abnormalities described in this report, such as cytopenias and liver function abnormalities, did not lead to clinically significant sequelae, possibly because of the close supervision of the clinical trial setting. It would, therefore, be prudent for clinicians to use similarly careful patient monitoring strategies for tocilizumab-treated patients in the clinical practice setting. Of note, however, the results of this integrated safety analysis are consistent overall with data from postmarketing surveillance to date.

## Conclusions

Results from the current analysis confirm that the longer-term safety profile of tocilizumab in RA patients is consistent with that previously described [1-5]. No new safety findings emerged in this large, pooled dataset with an extended treatment and observation period.

## Abbreviations

AE: adverse event; ALT: alanine aminotransferase; AMBITION: Actemra (Roche; Nutley: NJ: USA) versus Methotrexate double-Blind Investigative Trial In mONotherapy; ANC: absolute neutrophil count; AST: aspartate aminotransferase; BMI: body mass index; CI: confidence interval; CRP: C-reactive protein; CTC: Common Toxicity Criteria; CV: cardiovascular; DMARD: disease-modifying antirheumatic drug; GI: gastrointestinal; HDL: high-density lipoprotein; IL-6: interleukin-6; LDL: low-density lipoprotein; LITHE: tociLIzumab safety and THE prevention of structural joint damage; MedDRA: Medical Dictionary for Regulatory Activities; NSAID: nonsteroidal anti-inflammatory drug; OPTION: tOcilizumab Pivotal Trial in methotrexate Inadequate responders; PY: patient-year; RA: rheumatoid arthritis; RADIATE: Research on Actemra Determining efficacy after Anti-TNF failures; SAE: serious adverse event; TB: tuberculosis; TNF-α inhibitor: tumor necrosis factor inhibitor; TOWARD: Tocilizumab in cOmbination With traditional DMARD therapy; ULN: upper limit of normal.

## Competing interests

This study was funded by F. Hoffmann-La Roche Ltd. Michael H Schiff has served as consultant on advisory boards for Roche/Genentech. Joel M Kremer has received research grant support and consulting fees from Roche/Genentech and has served on the speakers' bureau for Roche/Genentech. Angelika Jahreis is an employee of Genentech. Emma Vernon is an employee of Roche. John D Isaacs has consulted for Roche and served on the Roche speakers' bureau. Ronald F van Vollenhoven has received research grant support and consulting fees from Roche/Genentech.

## Authors' contributions

All authors analyzed and interpreted the data, drafted or revised the manuscript critically for important intellectual content, and gave final approval of the version to be published.
